# The efficacy and safety of tislelizumab combined with weekly nab-paclitaxel, carboplatin, and cetuximab as first-line treatment for recurrent or metastatic head and neck squamous cell carcinoma: a real-world study

**DOI:** 10.3389/fimmu.2025.1708410

**Published:** 2026-01-09

**Authors:** Zhanyong Ouyang, Linting Zhang, Feng Wang, Meijing Chen, Boran Cheng, Fang Yang, Wenjuan Lai, Jing Gao, Shubin Wang, Gangling Tong

**Affiliations:** 1 Department of Oncology, Peking University Shenzhen Hospital Affiliated to Shenzhen University, Shenzhen, Guangdong, China; 2 Shenzhen Key Laboratory of Gastrointestinal Cancer Translational Research, Cancer Institute of Shenzhen-Peking University-Hong Kong University of Science and Technology (PKU-HKUST) Medical Center, Shenzhen, Guangdong, China; 3 Stomatological Center, Peking University Shenzhen Hospital, Shenzhen, Guangdong, China; 4 Nursing Department, Peking University Shenzhen Hospital, Shenzhen, Guangdong, China

**Keywords:** cetuximab, head and neck squamous cell carcinoma, immunotherapy, nab-paclitaxel, tislelizumab

## Abstract

**Background:**

Patients with recurrent or metastatic head and neck squamous cell carcinoma (R/M-HNSCC) face poor prognosis. This study evaluated the efficacy and safety of a novel first-line regimen combining tislelizumab, nab-paclitaxel, carboplatin, and cetuximab (TPCE) in R/M-HNSCC.

**Methods:**

In this retrospective study, 39 patients with R/M-HNSCC received tislelizumab (200 mg, day 1), nab-paclitaxel (125 mg/m^2^, days 1, 8), carboplatin (AUC = 2, days 1, 8), and cetuximab (400 mg/m^2^ loading dose, then 250 mg/m^2^ weekly, day 1) every 21 days for up to six cycles. Patients achieving stable disease or better continued maintenance therapy with cetuximab and tislelizumab until disease progression or unacceptable toxicity. The primary endpoint was objective response rate (ORR); secondary endpoints included disease control rate (DCR), duration of response (DOR), progression-free survival (PFS), overall survival (OS), and safety.

**Results:**

The TPCE regimen demonstrated significant antitumor activity, with an ORR of 82.1% and a DCR of 97.4%. With a median follow-up of 27.0 months (95% confidence interval [CI]: 23.6-30.4 months), the median DOR was 12.5 months (95% CI: 9.5-15.5 months), the median PFS was 14.0 months (95% CI: 11.0-17.1 months), and the median OS was 27.0 months (95% CI: 20.7-33.3 months). The 2-year PFS rate was 31.3 ± 7.8%, and the 2-year OS rate was 58.4 ± 8.4%. Subgroup analysis revealed a significantly higher ORR in patients with tongue carcinoma (P = 0.023). Lower baseline neutrophil-to-lymphocyte ratio (NLR, P = 0.044), systemic immune-inflammation index (SII, P = 0.044), and albumin level (P = 0.044) were correlated with improved ORR. A reduction in NLR after two treatment cycles was also associated with higher ORR (P = 0.037). Multivariate analysis identified baseline hemoglobin-albumin-lymphocyte-platelet (HALP) score as an independent prognostic factor for PFS (HR: 2.919; 95% CI: 1.153-7.391; P = 0.024), while primary tumor location (HR: 3.160; 95% CI: 1.205-8.282; P = 0.019), HALP score (HR: 3.541; 95% CI: 1.287-9.744; P = 0.014), and post-treatment SII changes (HR: 0.370; 95% CI: 0.151-0.906; P = 0.030) were independent predictors for OS. Grade 3–4 treatment-emergent adverse events were primarily hematologic, with granulocytopenia (38.5%) being the most common. Most immune-related adverse events were grade 1-2, with hypothyroidism (33.3%) occurring most frequently.

**Conclusion:**

The TPCE regimen demonstrated robust antitumor efficacy and a manageable safety profile as a first-line treatment for R/M-HNSCC. Baseline and dynamic inflammatory-nutritional markers may serve as predictive and prognostic indicators, supporting clinical decision-making for this patient population.

**Clinical trial registration:**

http://www.chictr.org.cn, identifier ChiCTR2500108877.

## 1 Introduction

Head and neck squamous cell carcinoma (HNSCC) comprises a group of highly invasive malignant tumors arising from the mucosal epithelium of the oral cavity, pharynx, larynx, and nasal cavity. As the seventh most common cancer worldwide, HNSCC accounts for approximately 890,000 new cases and 450,000 deaths annually, representing 4.5% of global cancer incidence and 4.6% of cancer-related mortality. Notably, over 60% of patients are diagnosed at a locally advanced stage (1). Despite the widespread use of multimodal treatments, including surgery, radiotherapy, and chemotherapy, the prognosis for HNSCC remains poor. Among patients with locally advanced disease, 40-60% eventually experience recurrence or metastasis, and the 5-year overall survival (OS) rate remains below 40%. For patients with recurrent or metastatic head and neck squamous cell carcinoma (R/M-HNSCC), the prognosis is even more grim, with a median survival typically less than one year (2). Hence, there is an urgent need for novel therapeutic strategies that can improve survival outcomes while maintaining acceptable toxicity profiles.

The therapeutic landscape for R/M-HNSCC has evolved significantly with the advent of targeted therapies and immunotherapies. The landmark EXTREME study was the first to demonstrate that cetuximab, combined with platinum-based chemotherapy and 5-fluorouracil, significantly improves outcomes in R/M-HNSCC, achieving an objective response rate (ORR) of 36%, with median progression-free survival (PFS) and OS of 5.6 months and 10.1 months, respectively (3). The subsequent GORTEC 2014–01 TPExtreme trial showed that the TPEx regimen (docetaxel, cisplatin, and cetuximab) offered similar efficacy compared to the EXTREME regimen (median OS: 14.5 vs. 13.4 months, P = 0.23), with improved safety profiles (4). The advent of immune checkpoint inhibitors (ICIs) has further revolutionized the treatment landscape for R/M-HNSCC. Four-year follow-up results from the KEYNOTE-048 study revealed similar PFS across the pembrolizumab monotherapy (programmed death-1 (PD-1) inhibitors), pembrolizumab plus chemotherapy, and cetuximab plus chemotherapy groups. However, OS differed significantly, with respective medians of 11.5, 13.0, and 10.7 months (P < 0.05). The ORRs were 16.9%, 36.3%, and 36.3% in the respective groups (5). Despite these advances, clinical outcomes remain suboptimal, with ORR typically below 40% in most treatment regimens. Moreover, there is still a significant unmet need, particularly in patients with low or negative programmed-death ligand 1 (PD-L1) expression. Therefore, the exploration of novel combination strategies to optimize treatment regimens continues to be a major focus of ongoing clinical research.

Emerging evidence has shown that cetuximab treatment in HNSCC can lead to the upregulation of PD-1 and T cell immunoglobulin and mucin domain protein-3 (TIM-3) expression in CD8^+^ tumor-infiltrating lymphocytes (TILs) within the tumor microenvironment. This upregulation is associated with poor clinical outcomes, suggesting that combining cetuximab with ICIs may reverse immunosuppression (6). Clinical evidence has supported this theoretical hypothesis. In patients with R/M-HNSCC, the combination of pembrolizumab and cetuximab demonstrated promising antitumor activity, achieving an ORR of 45% and manageable toxicity, with grade 3–4 oral mucositis occurring in 9% of cases (7). Another study reported that nivolumab combined with cetuximab yielded a median OS of 11.4 months in 45 patients who had previously received systemic therapy, and 20.2 months in 43 treatment-naïve patients (8). In parallel, the combination of chemotherapy and ICIs has also shown encouraging results. A regimen of pembrolizumab with nab-paclitaxel and platinum agents in patients with R/M-HNSCC achieved an ORR of 62.7%, a median PFS of 9.7 months, and a median OS of 18.7 months, with hematologic toxicity being the primary adverse event (grade 3 neutropenia in 28.4% of patients) (9). Furthermore, it has been reported that tislelizumab, a PD-1 inhibitor, yielded an ORR of 50%, a median PFS of 6.44 months, and a median OS of 20.07 months in a retrospective cohort of patients with R/M-HNSCC (10). Notably, nab-paclitaxel, due to its unique advantages—such as not requiring steroid premedication and having a favorable safety profile—has emerged as a suitable agent for combination with ICIs. However, despite these advances, the clinical potential of combining tislelizumab with nab-paclitaxel, carboplatin, and cetuximab (TPCE regimen) as first-line treatment for R/M-HNSCC remains unexplored. Based on this rationale, this retrospective study of patients treated at Peking University Shenzhen Hospital aims to evaluate the efficacy and safety of the TPCE regimen as a first-line therapeutic option, and to explore its potential activity in comparison with historical data from other regimens.

## 2 Materials and methods

### 2.1 Study design and patient selection

**Figure 1:**
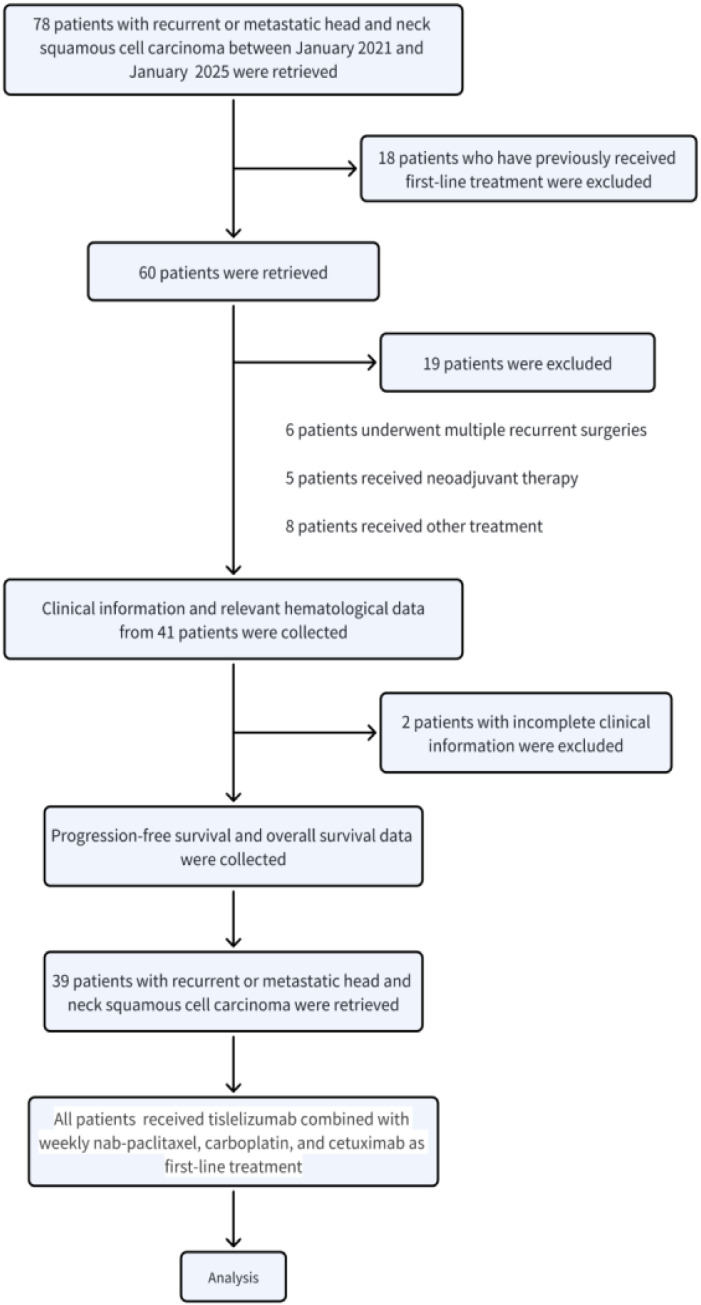
Trial profile.

This single-center, retrospective cohort study evaluated patients with histologically confirmed HNSCC treated at Peking University Shenzhen Hospital between January 2021 and January 2025. The study population comprised 39 patients meeting stringent selection criteria (**Figure 1**). Inclusion criteria: Patients were eligible if they fulfilled all of the following conditions: 1) Histologically confirmed squamous cell carcinoma originating from the oral cavity, oropharynx, hypopharynx, or larynx; 2) Diagnosis of R/M-HNSCC; 3) Treatment-naïve for R/M disease (first-line setting); 4) Age between 18 and 85 years; 5) Eastern cooperative oncology group performance status (ECOG PS) of 0-1; 6) Adequate organ function, defined as: Hematologic: absolute neutrophil count (ANC) ≥ 1.5×10^9^/L, platelet count ≥ 100×10^9^/L; Hepatic: total bilirubin ≤ 1.5 × upper limit of normal (ULN), aspartate aminotransferase (AST) and alanine aminotransferase (ALT) ≤ 2.5 × ULN; Renal: serum creatinine ≤ 1.5 × ULN; 7) Disease not amenable to curative local therapy at the time of initial R/M-HNSCC diagnosis; 8) During the initial treatment phase of the patient, there must be at least one measurable lesion, as defined by RECIST version 1.1.; 9) Availability of complete clinical and medical records. Exclusion criteria: Patients were excluded if they met any of the following conditions: 1) Diagnosis of nasopharyngeal carcinoma, salivary gland tumors, or cutaneous malignancies; 2) R/M-HNSCC considered potentially curable with local therapy; 3) Disease progression occurring within 6 months of completing prior definitive therapy for locally advanced HNSCC; 4) Prior treatment with systemic chemotherapy or ICIs; 5) Known hypersensitivity or severe allergic reaction to any study drugs, including nab-paclitaxel, cetuximab, carboplatin, or tislelizumab; 6) Presence of central nervous system (CNS) metastases. We used the STROBE reporting guideline (11) to draft this manuscript, and the STROBE reporting checklist (12) when editing, included in the strobe-checklist. The study protocol was approved by the Institutional Review Board of Peking University Shenzhen Hospital ([2025] No. (044)) and conducted in accordance with the principles of the Declaration of Helsinki. The need for informed consent was waived due to the retrospective nature of the study.

### 2.2 Treatment

The TPCE combination regimen was administered to eligible patients with R/M-HNSCC in two distinct phases: induction and maintenance. Induction Phase: During the induction phase, patients received the following regimen every 21 days for a total of six cycles: Tislelizumab (BeiGene, Ltd.) at 200 mg via intravenous infusion on Day 1; nab-paclitaxel (CSPC Ouyi Pharmaceutical Co., Ltd.) at 125 mg/m^2^ via intravenous infusion on Days 1, 8; carboplatin (Hisun Pharmaceutical Co., Ltd.) at an area under the curve (AUC) of 2 via intravenous infusion on Days 1, 8; cetuximab (Merck KGaA, Germany) with an initial loading dose of 400 mg/m^2^ followed by a weekly maintenance dose of 250 mg/m^2^. Maintenance Phase: After completing six cycles of induction treatment, patients who achieved stable disease (SD) or better received maintenance therapy with cetuximab (250 mg/m^2^, weekly) in combination with tislelizumab (200 mg every 3 weeks). Maintenance therapy continued until radiologically confirmed disease progression (PD), the occurrence of intolerable adverse events, or withdrawal by the patient. Adverse reactions were meticulously documented during each treatment cycle and graded according to the National Cancer Institute Common Terminology Criteria for Adverse Events (CTCAE) version 5.0. This comprehensive monitoring ensured the safety and well-being of patients throughout the study.

### 2.3 Assessments

All enrolled patients with R/M-HNSCC underwent standardized follow-up until June 2025 or until death. The primary endpoint was ORR. Secondary endpoints included disease control rate (DCR), PFS, OS, and safety. Efficacy was assessed according to RECIST version 1.1. and was classified as complete response (CR), partial response (PR), SD or PD. All CR and PR were required to be confirmed by subsequent radiographic imaging performed at least 4 weeks after the initial response assessment. ORR was calculated as the proportion of patients achieving CR or PR, while DCR included patients with CR, PR, or SD. Computed tomography (CT) scans and magnetic resonance imaging (MRI) were performed every two cycles during induction phase. Evaluations were conducted every 2 months during maintenance therapy. Patients who discontinued treatment were followed up for survival on a quarterly basis. OS was defined as the time from the first day of systemic therapy to death from any cause or last follow-up. PFS was defined as the time from the first day of systemic therapy to the first occurrence of local recurrence and/or distant metastasis, or death from any cause, or the last follow-up. Duration of response (DOR) was defined as the time from the date of first documented response (which can be either CR or PR) to the date of first documented PD or death from any cause, whichever occurs first. Follow-up methods included outpatient visits, telephone calls, inpatient information inquiries, and other methods.

We also evaluated the correlation between nutritional and inflammatory indicators and efficacy and prognosis during treatment in R/M-HNSCC patients. Relevant indicators were collected before treatment and after two cycles of treatment. The indicators were defined as follows: NLR (neutrophil to lymphocyte ratio) = ratio of neutrophil count to lymphocyte count; NER (neutrophil to eosinophil ratio) = ratio of neutrophil count to eosinophil count; PLR (platelet to lymphocyte ratio) = ratio of platelet count to lymphocyte count; LMR (lymphocyte to monocyte ratio) = ratio of lymphocyte count to monocyte count; HALP (hemoglobin, albumin, lymphocyte and platelet score) = hemoglobin (g/L) × albumin (g/L) × lymphocyte count (10^9^/L)/platelet count (10^9^/L); PNI (prognostic nutritional index) = sum of albumin value (g/L) and 5 × lymphocyte count (10^9^/L); SII (systemic immune-inflammation index) = neutrophil count (10^9^/L) × platelet count (10^9^/L)/lymphocyte count (10^9^/L). Body mass index (BMI) was calculated by dividing weight (kg) by the square of height (m^2^). Patients were categorized into underweight (BMI < 18.5 kg/m^2^), normal weight (18.5 kg/m^2^ ≤ BMI ≤ 24.9 kg/m^2^), and overweight (BMI ≥ 25 kg/m^2^) groups based on World Health Organization standards. Additionally, the functional status of patients was assessed using ECOG PS.

### 2.4 Statistical analysis

All statistical analyzes were performed using SPSS statistical software (version 27.0.; IBM Corporation, USA), while data visualization was conducted using GraphPad Prism software (version 9.0.) In the efficacy correlation analysis, categorical variables were compared using the chi-square test or Fisher’s exact test (when the expected frequency was < 5). For continuous variables, the independent samples t-test was applied for normally distributed data, and the Mann-Whitney U test (Wilcoxon rank-sum test) was used for non-normally distributed data. The median value was used to determine cut-off points, and continuous variables were converted into categorical variables. Survival analysis was conducted using the Kaplan-Meier method to evaluate PFS and OS, with the Logrank test used for group comparisons. Prognostic factor analysis was performed using the Cox proportional hazards regression model for both univariate and multivariate analyzes. All tests were two-sided, and a P value of < 0.05 was considered statistically significant.

## 3 Results

### 3.1 Patients

A total of 39 patients with R/M-HNSCC meeting the inclusion and exclusion criteria were enrolled. The majority of the patients were middle-aged or younger, with 69.2% under 60 years old. Notably, 4 cases (10.3%) were aged over 80 years. Additionally, the cohort was predominantly male (71.8%) and had primary tongue carcinoma (51.3%). Pathological tissue from 36 patients was tested for PD-L1 combined positive score (CPS) using the 22C3 antibody. The results showed that 43.6% had PD-L1 CPS < 1, 48.7% had PD-L1 CPS ≥ 1, and 17.9% had PD-L1 CPS ≥ 20. The majority of patients (84.6%) were positive for epidermal growth factor receptor (EGFR) expression, while a higher proportion (76.9%) showed negative P16 expression. In terms of BMI, 61.5% of patients had a BMI between 18.5 and 24.9 kg/m^2^, and 23.1% had a BMI < 18.5 kg/m^2^. Various inflammatory and nutritional indicators (NLR, PLR, LMR, SII, HALP, etc.) were categorized into high and low groups based on the median values, with balanced distribution between groups (P > 0.05). Overall, this cohort represents a typical R/M-HNSCC population with well-balanced baseline characteristics, making it suitable for evaluating the efficacy of the TPCE regimen. The complete baseline characteristics are presented in **Table 1**.

**Table 1:** Patient characteristics at baseline (N = 39).

Characteristics	Number (%)
Age (years)	
< 60	27 (69.2)
≥ 60	12 (30.8)
Gender	
Male	28 (71.8)
Female	11 (28.2)
Location	
Tongue	20 (51.3)
Gingiva	8 (20.5)
Buccal mucosa	6 (15.4)
Other	5 (12.8)
ECOG PS	
0	20 (51.3)
1	19 (48.7)
PD-L1 (CPS)	
Negative (< 1)	17 (43.6)
Low expression (≥ 1 and < 20)	12 (30.8)
High expression (≥ 20)	7 (17.9)
Unknown	3 (7.70)
EGFR	
Negative	5 (12.8)
Positive	33 (84.6)
Unknown	1 (2.60)
P16	
Negative	30 (76.9)
Positive	8 (20.5)
Unknown	1 (2.60)
BMI (kg/m^2^)	
Underweight (< 18.5)	9 (23.1)
Normoweight (18.5-24.9)	24 (61.5)
Overweight (≥ 25)	6 (15.4)
Baseline indicator^a^	
NLR	
< 2.70	20 (51.3)
≥ 2.70	19 (48.7)
NER	
< 30.80	19 (48.7)
≥ 30.80	20 (51.3)
PLR	
< 224.05	19 (48.7)
≥ 224.05	20 (51.3)
LMR	
< 2.55	20 (51.3)
≥ 2.55	19 (48.7)
HALP	
< 23.74	19 (48.7)
≥ 23.74	20 (51.3)
SII	
< 629.3	19 (48.7)
≥ 629.3	20 (51.3)
GFR (ml/min/1.73m^2^)	
< 105.2	19 (48.7)
≥ 105.2	20 (51.3)
Albumin (g/L)	
< 39.1	20 (51.3)
≥ 39.1	19 (48.7)
Total protein (g/L)	
< 69.4	18 (46.2)
≥ 69.4	21 (53.8)
PNI	
< 43.8	20 (51.3)
≥ 43.8	19 (48.7)

ECOG PS, eastern cooperative oncology group performance status; PD-L1, programmed cell death protein-ligand 1; CPS, combined positive score; EGFR, epidermal growth factor receptor; P16, a tumor suppressor gene; BMI, body mass index; NLR, neutrophil count to lymphocyte count ratio; NER, neutrophil count to eosinophil count ratio; PLR, platelet count to lymphocyte count ratio; LMR, lymphocyte count to monocyte count ratio; HALP, the hemoglobin, albumin, lymphocyte and platelet score; GFR, glomerular filtration rate; SII, systemic immune-inflammation index; PNI, prognostic nutritional index.

a The baseline measures were divided into two categories according to the median value of the subjects working baseline characteristics.

### 3.2 Clinical response rate and influencing factors

Among the 39 patients with R/M-HNSCC, CR was observed in 6 cases (15.4%), PR in 26 cases (66.7%), SD in 6 cases (15.4%), and PD in 1 case (2.6%), resulting in an ORR of 82.1% (32/39) and a DCR of 97.4% (38/39). The median DOR was 12.5 months (95% confidence interval [CI]: 9.5-15.5 months), and the DOR for CR patients was 17 months (range 7.5-44.5 months). We analyzed the correlation between clinical characteristics and treatment efficacy. The primary tumor site was significantly associated with ORR. Patients with primary tumors located in the tongue exhibited better efficacy, with an ORR of 48.7%. In contrast, those with buccal mucosa carcinoma had the lowest efficacy, with an ORR of only 12.8% (P = 0.023). Although patients with PD-L1 CPS ≥ 1 had higher ORR and DCR (43.6% and 48.6%, respectively) compared to those with PD-L1 CPS < 1 (30.7% and 41.0%, respectively), these differences were not statistically significant (P = 0.296 for ORR, P = 0.999 for DCR). However, among the 7 patients with PD-L1 CPS ≥ 20, 3 achieved CR (42.9%) and 4 achieved PR (57.1%), resulting in both ORR and DCR of 100%. Similarly, patients who were EGFR-positive and P16-negative tended to have better efficacy than those who were EGFR-negative and P16-positive, although these differences were not statistically significant (P > 0.05). Patients with BMI between 18.5 and 24.9 also showed better efficacy than those with BMI < 18.5 and ≥ 25 (P > 0.05), however, there was no statistical difference. In summary, primary tumors located in the tongue and PD-L1 CPS ≥ 20 were associated with better treatment response. Detailed results are presented in **Table 2**.

**Table 2:** Subgroup analysis of ORR and DCR of patients (N = 39).

Responses characteristics	ORR N (%)	Non-ORR N (%)	*P* value (ORR)	DCR N (%)	Non-DCR N (%)	*P* value (DCR)
Age (years)			0.649			0.999
< 60	22 (56.4)	6 (15.4)		27 (69.2)	1 (2.60)	
≥ 60	10 (25.6)	1 (2.60)		11 (28.2)	0 (2.60)	
Gender			0.649			0.282
Male	22 (56.4)	6 (15.4)		28 (71.8)	0 (0.0)	
Female	10 (25.6)	1 (2.60)		10 (25.6)	1 (2.60)	
Location			0.023			0.279
Tongue	19 (48.7)	1 (2.60)		20 (51.3)	0 (0.0)	
Gingiva	6 (15.4)	2 (5.10)		8 (20.5)	0 (0.0)	
Buccal mucosa	5 (12.8)	1 (2.60)		5 (12.8)	1 (2.60)	
Other	2 (5.10)	3 (7.70)		5 (12.8)	0 (0.0)	
ECOG PS			0.695			0.487
0	17 (43.6)	3 (7.70)		20 (51.3)	0 (0.0)	
1	15 (38.5)	4 (10.3)		18 (46.2)	1 (2.60)	
PD-L1 (CPS)			0.296			0.999
Negative	12 (30.7)	5 (12.8)		16 (41.0)	1 (2.60)	
Low expression (≥ 1 and < 20)	10 (25.6)	2 (5.10)		12 (30.7)	0 (0.0)	
High expression (≥ 20)	7 (17.9)	0 (0.0)		7 (17.9)	0 (0.0)	
Unknown	3 (7.70)	0 (0.0)				
EGFR			0.999			0.999
Negative	4 (10.3)	1 (2.60)		5 (12.8)	0 (0.0)	
Positive	27 (69.2)	6 (15.4)		32 (82.1)	1 (2.60)	
Unknown	1 (2.60)	0 (0.0)				
P16			0.307			0.999
Negative	23 (59.0)	7 (17.9)		29 (74.4)	1 (2.60)	
Positive	8 (20.5)	0 (0.0)		8 (20.5)	0 (0.0)	
Unknown	1 (2.60)	0 (0.0)				
BMI (kg/m^2^)			0.433			0.999
Underweight (< 18.5)	7 (17.9)	2 (5.10)		9 (23.1)	0 (0.0)	
Normoweight (18.5-24.9)	21 (53.8)	3 (7.70)		23 (59.0)	1 (2.60)	
Overweight (≥ 25)	4 (10.3)	2 (5.10)		6 (15.4)	0 (0.0)	
NLR			0.044			0.999
< 2.70	19 (48.7)	1 (2.60)		19 (48.7)	1 (2.60)	
≥ 2.70	13 (33.3)	6 (15.4)		19 (48.7)	0 (0.0)	
NER			0.405			0.486
< 30.80	16 (41.0)	2 (5.10)		17 (43.6)	1 (2.60)	
≥ 30.80	16 (41.0)	5 (12.8)		21 (53.8)	0 (0.0)	
PLR			0.695			0.487
< 224.05	15 (38.5)	4 (10.3)		18 (46.2)	1 (2.60)	
≥ 224.05	17 (43.6)	3 (7.70)		20 (51.3)	0 (0.0)	
LMR			0.999			0.487
< 2.55	16 (41.0)	4 (10.3)		20 (51.3)	0 (0.0)	
≥ 2.55	16 (41.0)	3 (7.70)		18 (46.2)	1 (2.60)	
HALP			0.695			0.487
< 23.74	17 (43.6)	3 (7.70)		20 (51.3)	0 (0.0)	
≥ 23.74	15 (38.5)	4 (10.3)		18 (46.2)	1 (2.60)	
SII			0.044			0.999
< 629.3	19 (48.7)	1 (2.60)		19 (48.7)	1 (2.60)	
≥ 629.3	13 (33.3)	6 (15.4)		19 (48.7)	0 (0.0)	
GFR (ml/min/1.73m^2^)			0.215			0.462
< 105.2	19 (48.7)	2 (5.10)		21 (53.8)	0 (0.0)	
≥ 105.2	13 (33.3)	5 (12.8)		17 (43.6)	1 (2.60)	
Albumin (g/L)			0.044			0.499
< 39.1	19 (48.7)	1 (2.60)		20 (51.3)	0 (0.0)	
≥ 39.1	13 (33.3)	6 (15.4)		18 (46.2)	1 (2.60)	
Total protein (g/L)			0.999			0.487
< 69.4	16 (41.0)	4 (10.3)		20 (51.3)	0 (0.0)	
≥ 69.4	16 (41.0)	3 (7.70)		18 (46.2)	1 (2.60)	
PNI			0.235			0.487
< 43.8	18 (46.2)	2 (5.10)		20 (51.3)	0 (0.0)	
≥ 43.8	14 (35.9)	5 (12.8)		18 (46.2)	1 (2.60)	

ORR, objective response rate; DCR, disease control rate; ECOG PS, eastern cooperative oncology group performance status; PD-L1, programmed cell death protein-ligand 1; CPS, combined positive score; EGFR, epidermal growth factor receptor; P16, a tumor suppressor gene; BMI, body mass index; NLR, neutrophil count to lymphocyte count ratio; NER, neutrophil count to eosinophil count ratio; PLR, platelet count to lymphocyte count ratio; LMR, lymphocyte count to monocyte count ratio; HALP, the hemoglobin, albumin, lymphocyte and platelet score; GFR, glomerular filtration rate; SII, systemic immune-inflammation index; PNI, prognostic nutritional index.

Furthermore, we investigated the relationship between nutritional and inflammatory markers and clinical efficacy during treatment. Patients with a pre-treatment NLR < 2.70 exhibited a significantly higher ORR compared to those with NLR ≥ 2.70 (48.7% vs. 33.3%, P = 0.044). Similarly, patients with a pre-treatment SII < 629.3 had a better ORR than those with SII ≥ 629.3 (48.7% vs. 33.3%, P = 0.044). In addition, ORR was better in Albumin < 39.1 than in Albumin ≥ 39.1 (48.7% vs. 33.3%, P = 0.044). Details are provided in **Table 2**. We also evaluated the association between these markers and efficacy after 2 treatment cycles. Patients with decreased post-treatment NLR showed significantly higher ORR compared to those with increased NLR (69.2% vs. 12.8%, P = 0.037). Other nutritional and inflammatory indicators did not show a significant correlation with ORR or DCR (P > 0.05). In summary, lower baseline levels of NLR, SII, and albumin were associated with higher ORR. Additionally, patients with decreased NLR after two treatment cycles had better response rates. Detailed results are presented in **Table 3**.

**Table 3:** Subgroup analysis of ORR and DCR following 2 cycles of treatment of patients (N = 39).

Responses characteristics	ORR N (%)	Non-ORR N (%)	*P* value (ORR)	DCR N (%)	Non-DCR N (%)	*P* value (DCR)
NLR			0.037			0.231
Decrease	27 (69.2)	3 (7.70)		30 (76.9)	0 (0.0)	
Increase	5 (12.8)	4 (10.3)		8 (20.5)	1 (2.60)	
NER			0.674			0.432
Decrease	14 (37.8)	2 (5.40)		15 (40.5)	1 (2.70)	
Increase	16 (43.2)	5 (13.5)		21 (56.8)	0 (0.0)	
PLR			0.410			0.410
Decrease	23 (59.0)	0 (0.0)		23 (59.0)	0 (0.0)	
Increase	15 (38.5)	1 (2.60)		15 (38.5)	1 (2.60)	
LMR			0.999			0.359
Decrease	12 (30.8)	2 (5.10)		13 (33.3)	1 (2.60)	
Increase	20 (51.3)	5 (12.8)		25 (64.1)	0 (0.0)	
HALP			0.678			0.999
Decrease	18 (46.2)	5 (12.8)		22 (56.4)	1 (2.60)	
Increase	14 (35.9)	2 (5.10)		16 (41.0)	0 (0.0)	
SII			0.686			0.359
Decrease	21 (53.8)	4 (10.3)		25 (64.1)	0 (0.0)	
Increase	11 (28.2)	3 (7.70)		13 (33.3)	1 (2.60)	
ALB			0.215			0.462
Decrease	13 (33.3)	5 (12.8)		17 (43.6)	1 (2.60)	
Increase	19 (48.7)	2 (5.10)		21 (53.8)	0 (0.0)	
PNI			0.686			0.359
Decrease	11 (28.2)	3 (7.70)		13 (33.3)	1 (2.60)	
Increase	21 (53.8)	4 (10.3)		25 (64.1)	0 (0.0)	
BMI			0.653			0.999
Decrease	25 (64.1)	5 (12.8)		29 (74.4)	1 (2.60)	
Increase	7 (17.9)	2 (5.10)		9 (23.1)	0 (0.0)	

ORR, objective response rate; DCR, disease control rate; NLR, neutrophil count to lymphocyte count ratio; NER, neutrophil count to eosinophil count ratio; PLR, platelet count to lymphocyte count ratio; LMR, lymphocyte count to monocyte count ratio; HALP, the hemoglobin, albumin, lymphocyte and platelet score; GFR, glomerular filtration rate; SII, systemic immune-inflammation index; ALB, albumin; PNI, prognostic nutritional index; BMI, body mass index.

### 3.3 Clinical prognosis and influencing factors

**Figure 2:**
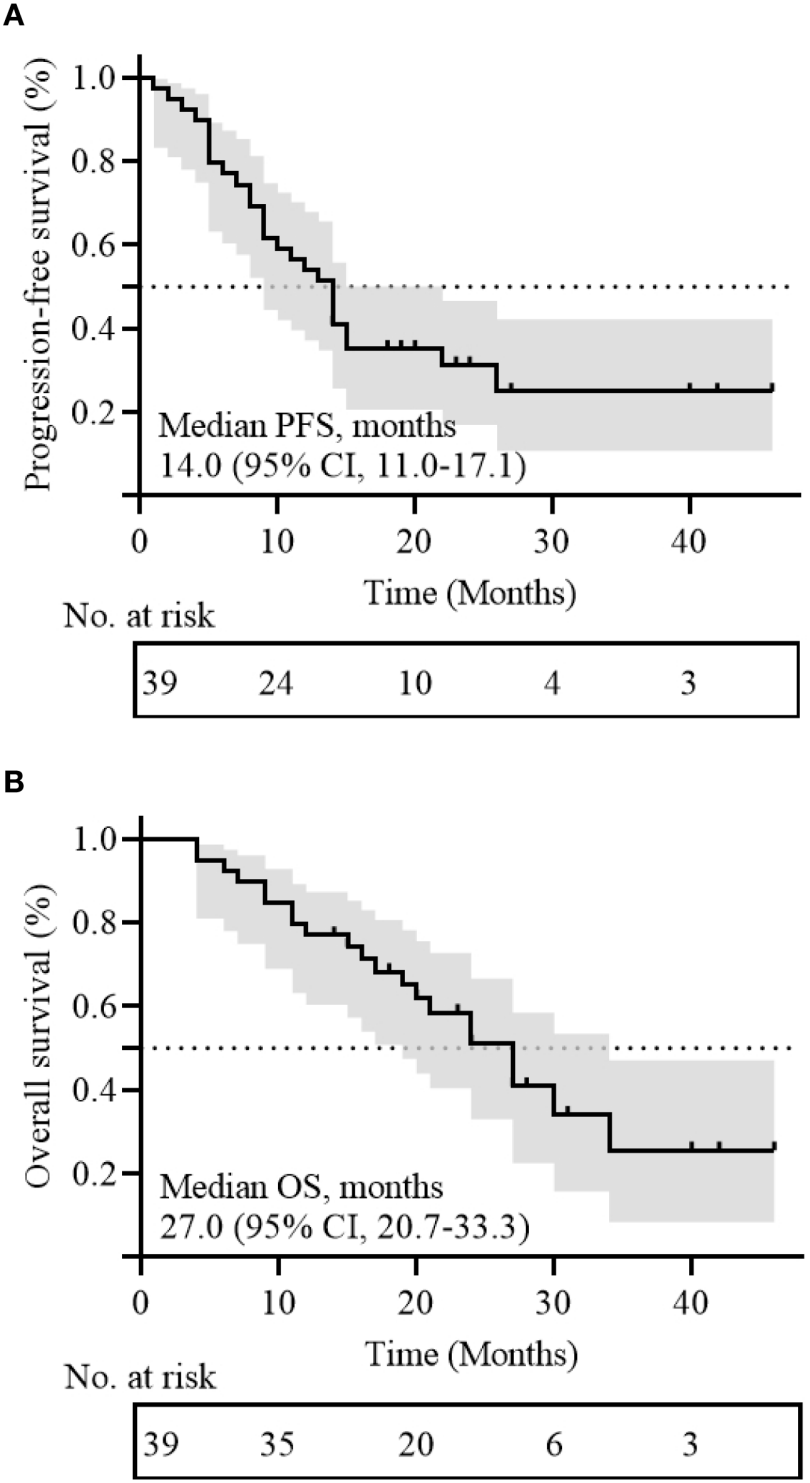
Kaplan-Meier curves of **(A)** progression free survival (PFS) and **(B)** overall survival (OS) for all patients; CI, confidence interval.

**Figure 3:**
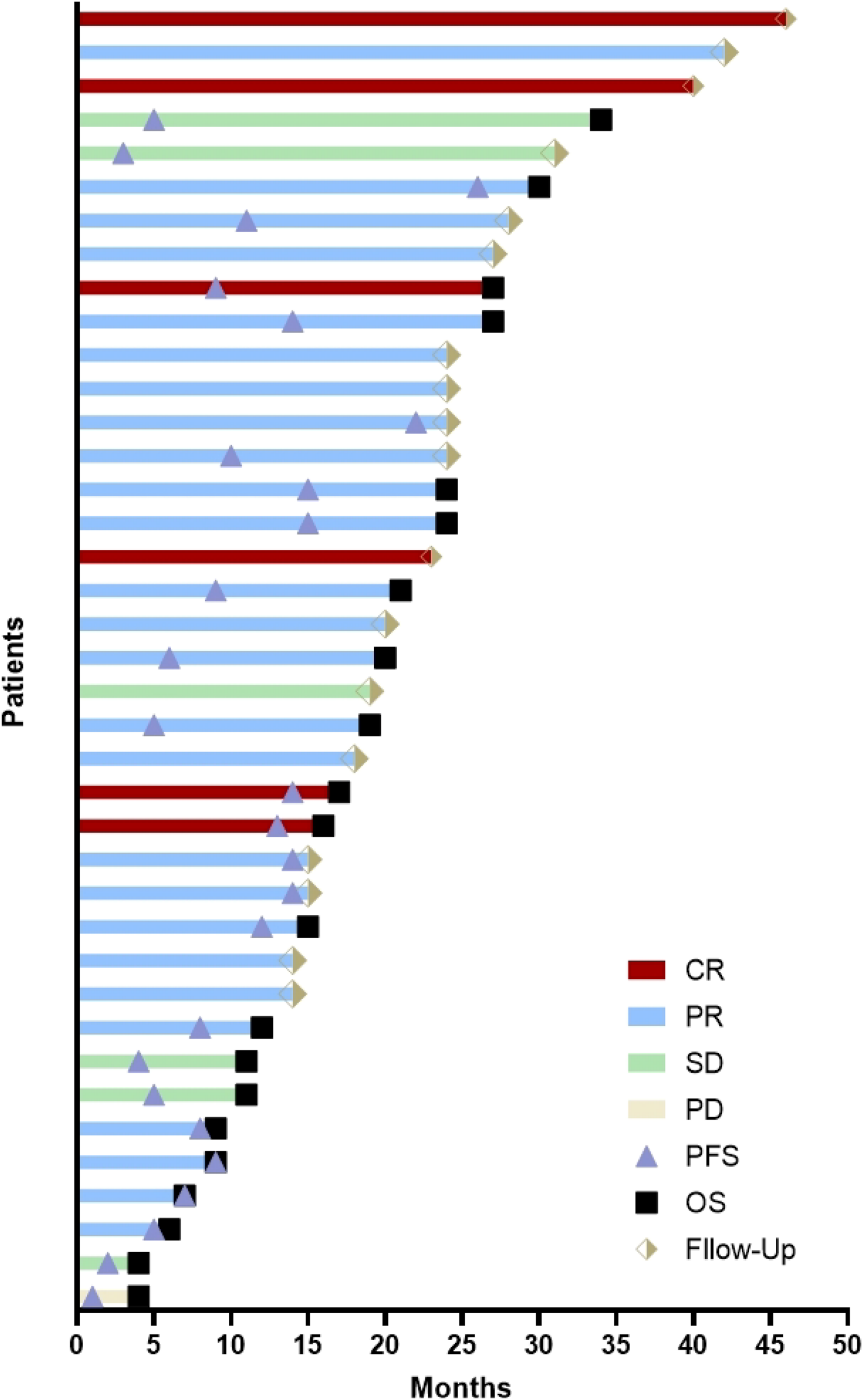
A swimmer’s plot of all patients. Abbreviation used: CR, complete response; PR, partial response; SD, stable disease; PD, disease progression; PFS, progression-free survival; OS, overall survival.

As of the follow-up cutoff, the median follow-up time for the 39 patients with R/M-HNSCC was 27.0 months (95% CI: 23.6-30.4 months). The median PFS was 14.0 months (95% CI: 11.0-17.1 months; **Figure 2A**), the median OS was 27.0 months (95% CI: 20.7-33.3 months; **Figure 2B**), and the median DOR was 12.5 months (95% CI: 9.5-15.5 months). The 2-year PFS rate was 31.3 ± 7.8%, and the 2-year OS rate was 58.4 ± 8.4%. A swimmer’s plot is used to visually represent the response dynamics and duration for all patients (**Figure 3**).

**Figure 4:**
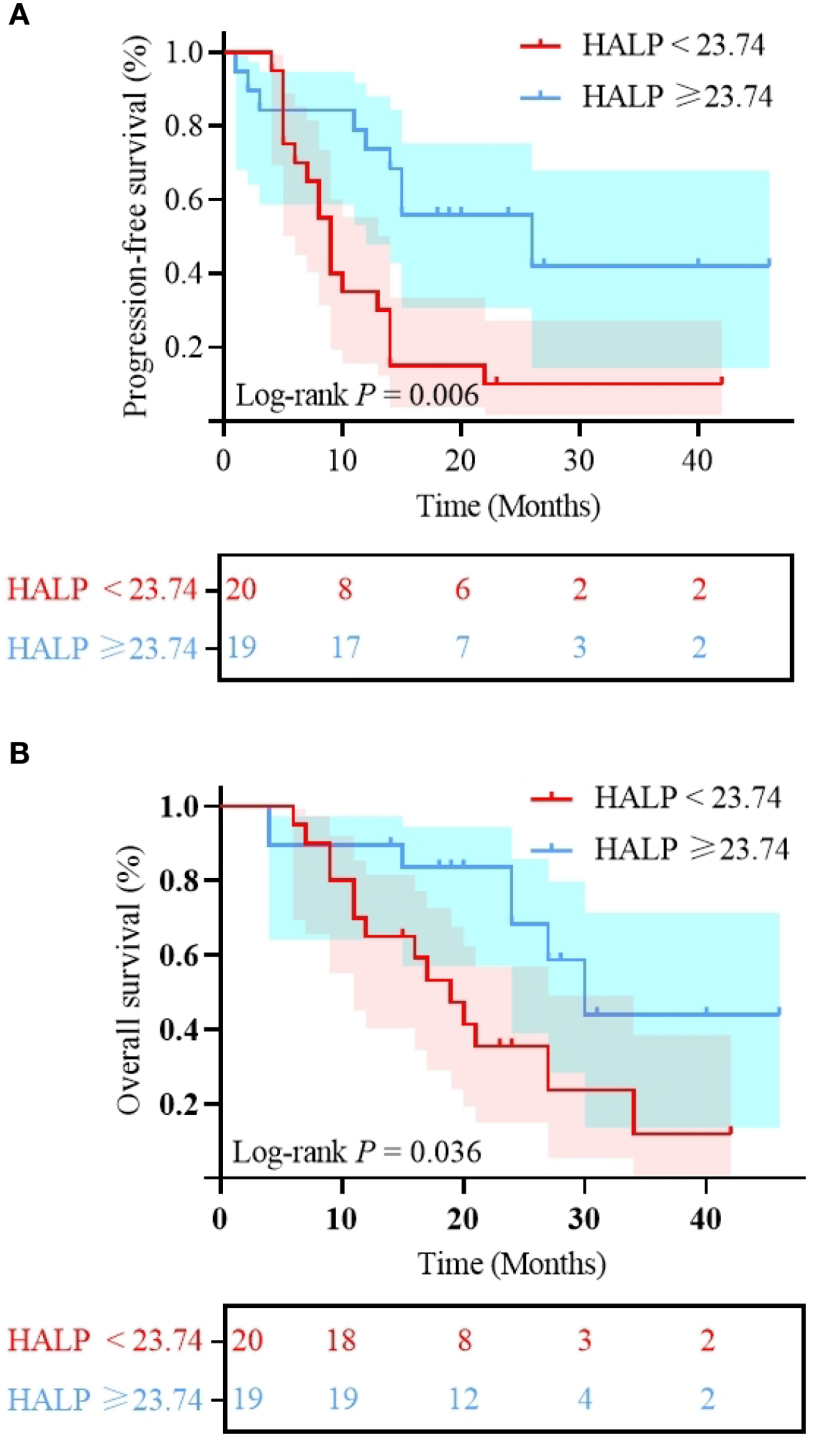
Kaplan-Meier curves of **(A)** progression free survival stratified by HALP; **(B)** Overall survival stratified by HALP; HALP, the hemoglobin, albumin, lymphocyte and platelet score.

**Figure 5:**
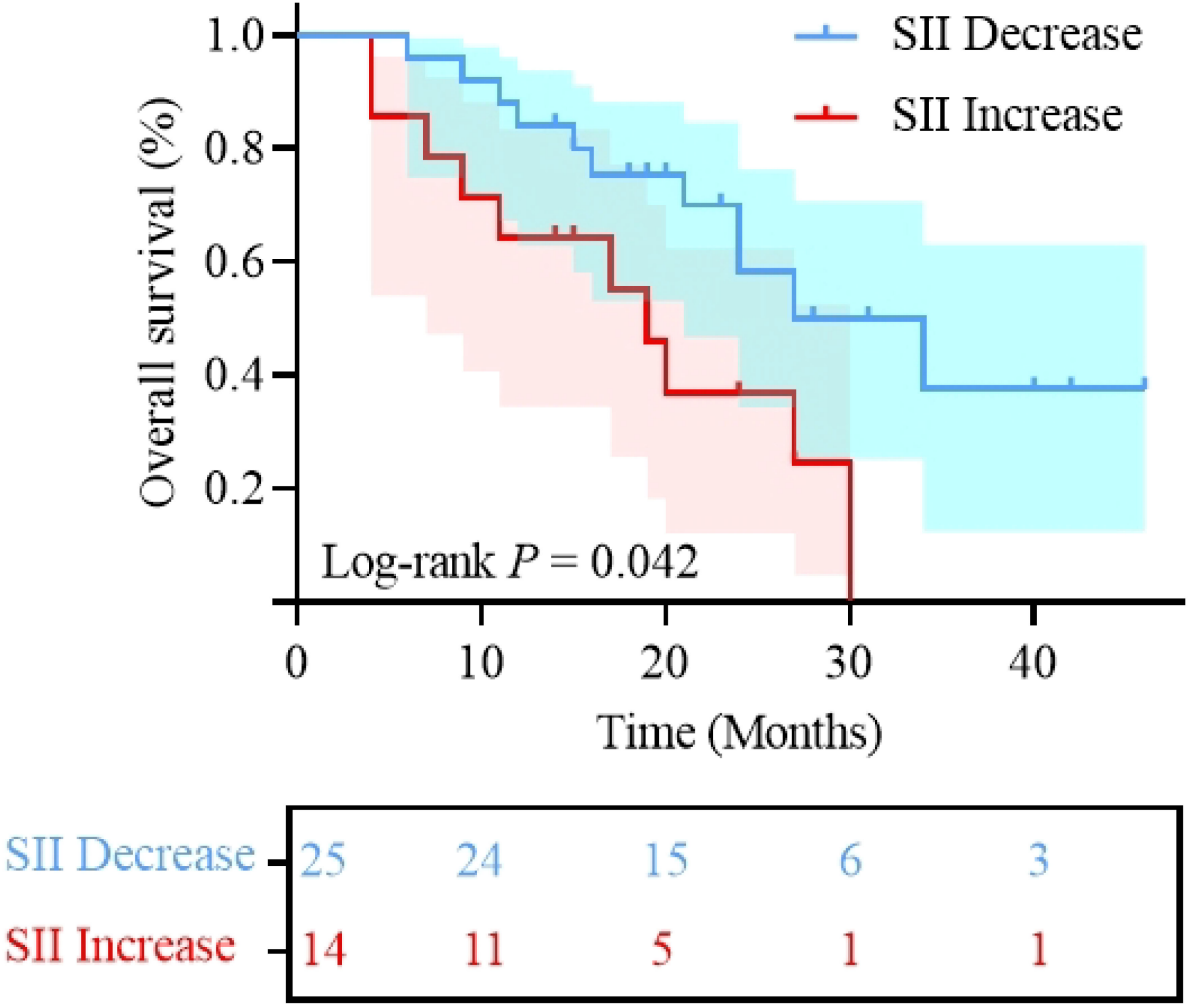
Kaplan-Meier curves of overall survival stratified by post-treatment SII changes (Decrease *vs.* Increase); SII, systemic immune-inflammation index.

**Figure 6:**
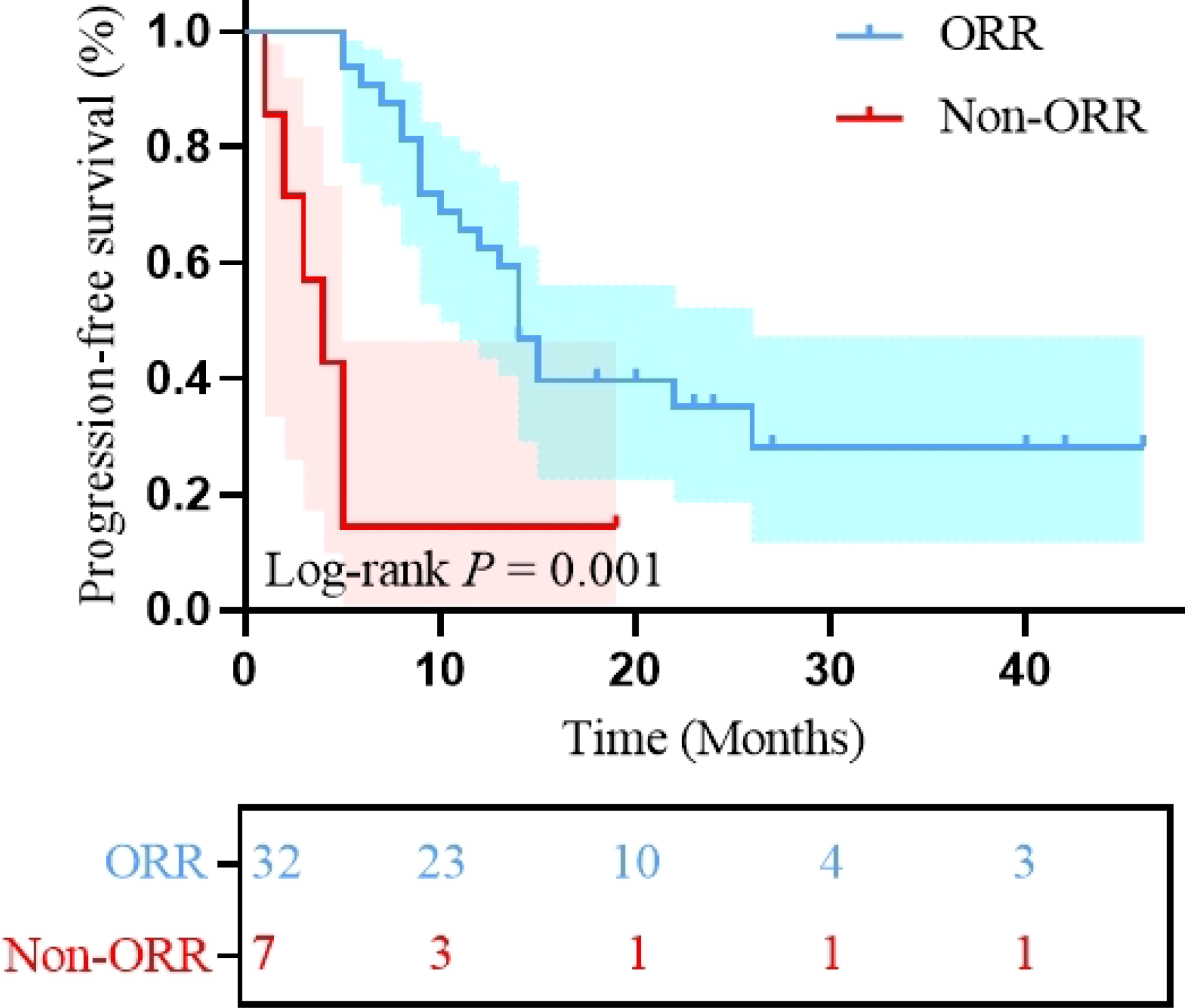
Kaplan-Meier curves of progression free survival stratified by for treatment efficacy (ORR *vs.* Non-ORR); ORR: objective response rate.

We analyzed the association between clinical characteristics, nutritional and inflammatory markers before treatment and after 2 cycles, and clinical prognosis using univariate analysis. Univariate analysis revealed that HALP (< 23.74 *vs*. ≥ 23.74; HR: 3.189; 95% CI: 1.401-7.257, P = 0.006; **Figure 4A**) was significantly associated with PFS. For OS, significant predictors included HALP (< 23.74 *vs*. ≥ 23.74; HR: 2.675; 95% CI: 1.067-6.707; P = 0.036; **Figure 4B**) and post-treatment SII changes (Decrease *vs*. Increase; HR: 0.401; 95% CI: 0.166-0.969; P = 0.042; **Figure 5**). Additionally, this study indicated a significant correlation between PFS and treatment efficacy (ORR *vs.* Non-ORR; HR: 0.216; 95% CI: 0.084-0.556; P = 0.001; **Figure 6**), meaning better efficacy associated with improved PFS. Given the sample size constraints, the following multivariable analysis should be considered exploratory. To avoid overfitting, the number of variables included in the Cox multivariate regression was limited based on the sample size. First, univariate Cox regression identified three variables with a trend toward significance for PFS (P < 0.1): BMI, HALP, and SII; and three variables for OS (P < 0.05): location, HALP, and post-treatment SII changes. These variables were then included in multivariate Cox regression analysis. The results showed that HALP (HR: 2.919; 95% CI: 1.153-7.391; P = 0.024) was an independent prognostic factor for PFS. For OS, location (HR: 3.160; 95% CI: 1.205-8.282; P = 0.019), HALP (HR: 3.541; 95% CI: 1.287-9.744; P = 0.014), and post-treatment SII changes (HR: 0.370; 95% CI: 0.151-0.906; P = 0.030) were independent prognostic factors. Other indicators did not significantly affect clinical prognosis. Detailed results are presented in **Table 4**. In summary, primary tumors located in the non-tongue, pre-treatment HALP < 23.74 and increased post-treatment SII were identified as unfavorable prognostic factors.

**Table 4:** Univariate and multivariate analyses of PFS and OS of patients (N = 39).

Variables	PFS				OS			
	Univariate		Multivariate		Univariate		Multivariate	
	HR (95% CI)	*P* value	HR (95% CI)	*P* value	HR (95% CI)	*P* value	HR (95% CI)	*P* value
Gender	0.694 (0.309-1.557)	0.376			1.354 (0.532-3.447)	0.525		
Male *vs*. Female								
Location	1.069 (0.496-2.303)	0.865			2.562 (0.990-6.629)	0.052	3.160 (1.205-8.282)	0.019
Non-tongue *vs*. Tongue								
ECOG PS	1.046 (0.483-2.263)	0.909			1.257 (0.491-3.216)	0.633		
0 *vs*. 1								
PD-L1	0.975 (0.441-2.157)	0.950			0.769 (0.308-1.922)	0.574		
Negative *vs*. Positive								
EGFR	1.009 (0.302-3.375)	0.988			1.995 (0.556-7.163)	0.290		
Negative *vs*. Positive								
P16	1.025 (0.411-2.557)	0.958			1.173 (0.393-3.498)	0.775		
Negative *vs*. Positive								
BMI	2.122 (0.935-4.819)	0.072	0.984 (0.366-2.640)	0.974	1.207 (0.467-3.119)	0.698		
18.5-24.9 *vs*. Else								
NLR	0.692 (0.324-1.478)	0.341			1.484 (0.617-3.571)	0.378		
< 2.70 *vs*. ≥ 2.70								
NER	0.505 (0.225-1.135)	0.098			0.965 (0.395-2.357)	0.937		
< 30.80 *vs*. ≥ 30.80								
PLR	0.555 (0.255-1.207)	0.137			0.657 (0.275-1.566)	0.373		
< 224.05 *vs*. ≥ 224.05								
HALP	3.189 (1.401-7.257)	0.006	2.919 (1.153-7.391)	0.024	2.675 (1.067-6.707)	0.036	3.541 (1.287-9.744)	0.014
< 23.74 *vs*. ≥ 23.74								
SII	0.494 (0.229-1.066)	0.072	0.606 (0.257-1.431)	0.253	0.807 (0.342-1.903)	0.624		
< 629.3 *vs*. ≥ 629.3								
GFR	0.601 (0.281-1.286)	0.190			0.931 (0.395-2.196)	0.871		
< 105.2 *vs*. ≥ 105.2								
ALB	0.516 (0.240-1.108)	0.090			0.828 (0.349-1.966)	0.669		
< 39.1 *vs*. ≥ 39.1								
HALP	1.063 (0.495-2.282)	0.875			1.262 (0.532-2.993)	0.597		
Decrease *vs*. Increase								
SII	0.501 (0.233-1.078)	0.077			0.401 (0.166-0.969)	0.042	0.370 (0.151-0.906)	0.030
Decrease *vs*. Increase								

PFS, progression-free survival; OS, overall survival; HR, hazard ratio; CI, confidence interval; ECOG PS, eastern cooperative oncology group performance status; PD-L1, programmed cell death protein-ligand 1; EGFR, epidermal growth factor receptor; P16, a tumor suppressor gene; BMI, body mass index; NLR, neutrophil count to lymphocyte count ratio; NER, neutrophil count to eosinophil count ratio; PLR, platelet count to lymphocyte count ratio; HALP, the hemoglobin, albumin, lymphocyte and platelet score; SII, systemic immune-inflammation index; GFR, glomerular filtration rate; ALB, albumin.

### 3.4 Safety

Treatment emergent adverse event (TEAE) occurring during treatment in the 39 patients with R/M-HNSCC are detailed in **Table 5**. Adverse events of any grade were observed in all 39 patients (100%). Grade 3 adverse events were reported in 15 patients (38.5%), with neutropenia being the most common (20.5%). Other less frequent Grade 3 events included febrile neutropenia (2.70%), thrombocytopenia (5.10%), liver function injury (2.70%), anorexia (2.70%), rash (2.70%), and peripheral neurotoxicity (2.70%). Grade 4 adverse events occurred in 9 patients (23.1%), primarily neutropenia (17.9%), followed by febrile neutropenia (2.70%) and thrombocytopenia (2.70%). Potential immune-related adverse events included abnormal glucose tolerance in 1 patient (2.70%), hyperthyroidism in 1 patient (2.70%), hypothyroidism in 13 patients (33.3%), and pneumonia in 1 patient (2.70%); all were Grade 1-2. No treatment-related deaths were observed. Overall, the TPCE regimen was found to be safe as a first-line treatment for patients with R/M-HNSCC.

**Table 5:** Treatment emergent adverse event by grade.

TEAEs	Grade 1-2 (N,%)	Grade 3 (N,%)	Grade 4 (N,%)
Anemia	32 (82.1)	0 (0.0)	0 (0.0)
Peripheral neuropathy	26 (66.7)	1 (2.70)	0 (0.0)
Rash	26 (66.7)	1 (2.70)	0 (0.0)
Neutropenia	13 (33.3)	8 (20.5)	7 (17.9)
Anorexia	13 (33.3)	1 (2.70)	0 (0.0)
Hypothyroidism	13 (33.3)	0 (0.0)	0 (0.0)
Abnormal liver function	6 (15.4)	1 (2.70)	0 (0.0)
Fatigue	5 (12.8)	0 (0.0)	0 (0.0)
Mucositis	4 (10.3)	0 (0.0)	0 (0.0)
Thrombocytopenia	3 (7.70)	2 (5.10)	1 (2.70)
Nausea	3 (7.70)	0 (0.0)	0 (0.0)
Infusion reaction	2 (5.10)	0 (0.0)	0 (0.0)
Pneumonia	1 (2.70)	0 (0.0)	0 (0.0)
Abnormal glucose tolerance	1 (2.70)	0 (0.0)	0 (0.0)
Hyperthyroidism	1 (2.70)	0 (0.0)	0 (0.0)
Febrile neutropenia	0 (0.0)	1 (2.70)	1 (2.70)

TEAEs, Treatment emergent adverse events.

## 4 Discussion

HNSCC remains a significant global health challenge. There is a pressing need for more effective therapies, especially for patients with high tumor burden that critically impairs functions such as swallowing and speech, who require rapid tumor shrinkage (13). The treatment landscape for R/M-HNSCC has been reshaped by ICIs. Agents such as pembrolizumab and nivolumab have demonstrated efficacy in clinical trials like KEYNOTE-048 and CheckMate-141 and are now established treatment options (14). Nonetheless, response rates remain suboptimal. In KEYNOTE-048, the ORR for pembrolizumab plus chemotherapy was 37.0%, comparable to the EXTREME regimen’s 36.3%, while pembrolizumab monotherapy yielded an ORR of only 16.9% (15). Similarly, nivolumab achieved an ORR of 13.3% in the second-line setting (16). These figures underscore the need for more effective strategies.

Combining ICIs with EGFR inhibitors represents a promising approach via dual mechanisms of action (17). In comparison to the historical data from the II trial of cetuximab plus durvalumab reported an ORR of 39% in metastatic HNSCC (18). This study evaluated the efficacy of the TPCE regimen as a first-line treatment for patients with R/M-HNSCC. The results demonstrated a high ORR and DCR of 82.1% and 97.4%, respectively, which are numerically higher than the response rates reported in some previous clinical studies of other regimens. For contextual purposes, the single-arm Phase IV KEYNOTE-B10 clinical trial, which investigated first-line pembrolizumab combined with carboplatin and paclitaxel in R/M-HNSCC, reported an ORR of 49% and a DCR of 75%, with an ORR of 44% in patients with PD-L1 CPS ≥ 20 (19). Notably, in our cohort, all seven patients with PD-L1 CPS ≥20 achieved a response (ORR and DCR 100%), suggesting that high PD-L1 expression may identify a subgroup that derives particular benefit from the TPCE regimen, consistent with other findings. However, this correlation was not statistically significant, likely due to the limited sample size, and warrants further validation. The promising activity observed with the TPCE regimen in this study may be explained by several potential synergistic mechanisms. Beyond the potential synergy between tislelizumab and cetuximab, tislelizumab’s optimized Fc region reduces binding to Fcγ receptors on macrophages, potentially minimizing antibody-dependent cellular phagocytosis (ADCP) and enhancing anti-tumor activity (20). Furthermore, the inclusion of nab-paclitaxel may improve drug delivery and avoids the potential immunosuppressive effects of steroid premedication required with solvent-based taxanes (21). In summary, the TPCE regimen showed promising antitumor activity in this retrospective cohort, warranting further investigation in prospective studies.

Notably, the ORR among patients with primary tumors originating from the tongue was significantly higher at 48.7%, whereas patients with buccal mucosa carcinoma had an ORR of only 12.8% (P = 0.023). This difference may be related to the immune microenvironment or drug distribution characteristics of tongue tumors, and further research is needed to confirm this finding. We also identified potential biomarkers for treatment response. Lower baseline levels of NLR, SII, and albumin were associated with a higher ORR. A decrease in NLR after two treatment cycles was significantly correlated with improved response, and a similar trend was observed for SII reduction. These findings align with studies linking low NLR and SII to better outcomes in ICI-treated R/M-HNSCC and non-small cell lung cancer (NSCLC) (22, 23), although some reports on albumin’s predictive role have been inconsistent (24). The association between NLR reduction and improved efficacy has also been noted in advanced hypopharyngeal carcinoma and gastric cancer treated with chemoimmunotherapy (25, 26). Thus, baseline inflammatory-nutritional status and its early on-treatment dynamics appear to be associated with treatment efficacy in patients receiving the TPCE regimen, suggesting a potential link between systemic inflammation and immune response. However, the causal mechanisms underlying this relationship remain speculative.

The survival outcomes observed with the TPCE regimen compare favorably with existing therapies. The median PFS was 14.0 months and median OS was 27.0 months, respectively. The median DOR was 12.5 months. The 2-year PFS rate was 31.3 ± 7.8%, and the 2-year OS rate was 58.4 ± 8.4%. While cross-trial comparisons should be interpreted with caution, the median PFS and OS in our cohort appears numerically favorable to that reported in the CHANGE-2 trial (median PFS 5.5 months, OS 11.1 months) with a PF-cetuximab regimen (27), and a real-world study of taxane-ICI combinations (2-year PFS 34.4%, OS 36.9%) (28). The enhanced efficacy may be attributed to several mechanisms: nab-paclitaxel/carboplatin may potentiate the immune response by enhancing PD-L1 expression and CD8^+^ T-cell infiltration, while cetuximab can induce antibody-dependent cellular cytotoxicity and further upregulate PD-L1 (29), synergizing with tislelizumab. Prognostic analysis further underscored the importance of inflammatory-nutritional status. A lower pre-treatment HALP score was independently associated with inferior PFS and OS, consistent with findings in melanoma patients treated with nivolumab (30, 31). Similarly, multivariate analysis identified age, tumor location, and oral habits as independent factors influencing the survival of patients with oral squamous cell carcinoma. Specifically, tongue squamous cell carcinoma was associated with a more favorable prognosis relative to non-tongue carcinomas (32). Moreover, an increase in SII after two treatment cycles independently predicted poorer OS, aligning with evidence from hepatocellular carcinoma and NSCLC that rising systemic inflammation post-ICI initiation correlates with worse survival (33, 34). These results suggest that baseline HALP and alongside on-treatment SII dynamics, may serve as prognostic biomarkers, potentially reflecting the interplay between host nutritional status and systemic inflammation in shaping the tumor immune microenvironment (35). This supports the speculative notion that patients might benefit from nutritional support or inflammation modulation, though interventional studies are needed to confirm this hypothesis.

The TPCE regimen demonstrated a manageable safety profile as first-line treatment for R/M-HNSCC. Grade 3–4 treatment emergent adverse events (TEAEs) were predominantly hematologic, with neutropenia (38.5%) and thrombocytopenia (7.80%) being the most common. Immune-related adverse events (AEs) were primarily mild to moderate, with hypothyroidism (33.3%) as the most frequent event, and only one case each of glucose intolerance and hyperthyroidism was observed. Notably, no treatment-related deaths occurred. A subgroup analysis was performed on the four patients (10.3%) aged over 80 years. While the incidence of Grade 3–4 neutropenia was 25% (1/4), no Grade 4 non-hematologic toxicities or treatment-related deaths were observed. Dose modifications (reductions or delays) were required in 75% (3/4) of these elderly patients, primarily for hematologic toxicity. Despite these adjustments, all four patients completed induction therapy, with an ORR of 75% and a DCR of 100%, indicating that the regimen remained effective and manageable in this population with close monitoring. The hematologic toxicity profile of TPCE appears moderately higher than that reported for pembrolizumab combined with nab-paclitaxel/platinum (neutropenia: 28.4% vs. 38.5% in our study) (9), yet remains favorable compared to traditional tri-weekly paclitaxel/platinum regimens (neutropenia rates up to 49%) (36). This difference may be attributed to our weekly dosing schedule of nab-paclitaxel and carboplatin, which appears to reduce hematologic toxicity while maintaining efficacy. With its manageable TEAEs profile and significant antitumor activity, the TPCE regimen represents a promising first-line therapeutic option for R/M-HNSCC. These findings warrant further validation in larger prospective studies to optimize dosing strategies and long-term safety monitoring.

This study has several limitations that warrant consideration. First, the most important limitation is the relatively small sample size. Therefore, the associations identified as independent prognostic factors are considered hypothesis-generating and require confirmation in larger studies. Second, its single-center, retrospective design and modest sample size (N = 39) may introduce selection bias, potentially limiting the generalizability of the results to broader R/M-HNSCC populations. Third, PD-L1 expression was not assessed in all patients (36/39), and key biomarkers such as human papillomavirus (HPV) status were not systematically evaluated, restricting the ability to explore their prognostic or predictive roles. Fourth, the lack of long-term toxicity follow-up precludes a comprehensive assessment of the regimen’s safety profile over extended periods.

Despite these constraints, our findings provide compelling preliminary evidence supporting the TPCE regimen as a viable first-line option for R/M-HNSCC. The observed efficacy and favorable tolerability underscore the need for further validation in larger, prospective studies with biomarker-integrated analyzes.

## 5 Conclusion

Tislelizumab combined with weekly nab-paclitaxel, carboplatin, and cetuximab exhibited significant anti-tumor efficacy and a manageable safety profile in patients with R/M-HNSCC. Notably, higher ORRs were observed in subgroups with primary tongue tumors, PD-L1 CPS ≥ 20, and low baseline levels of NLR, SII, and ALB. Dynamic decrease in NLR after 2 cycles of treatment was associated with improved treatment response. Multivariate analysis identified pre-treatment HALP score, primary tumors located and post-treatment SII changes as independent prognostic factors. These findings suggest that the TPCE regimen is a promising therapeutic strategy worthy of further investigation in R/M-HNSCC and the associations between inflammatory-nutritional markers and outcomes highlight the potential role of host immunity and metabolic status in treatment response, though the mechanistic basis remains speculative and merits further study.

## Data Availability

The original contributions presented in the study are included in the article/**Supplementary Material**. Further inquiries can be directed to the corresponding author.
